# Cow’s Milk Protein Allergy, a Systematic Review of Clinical Characteristics, Diagnosis, Management, and Economic Impact

**DOI:** 10.3390/diseases14040146

**Published:** 2026-04-17

**Authors:** Fabiola Menco Contreras, Karina Pastor-Sierra, Nany Castilla Herrera

**Affiliations:** 1 Facultad de Ciencias de la Salud, Universidad del Sinú E.B.Z., Montería 230001, Colombia; fabiolamenco@unisinu.edu.co (F.M.C.); nanycastilla@unisinu.edu.co (N.C.H.); 2 Grupo de Investigación Biomédicas y Biología Molecular, Facultad de Ciencias de la Salud, Universidad del Sinú E.B.Z., Montería 230001, Colombia

**Keywords:** cow’s milk protein allergy, food allergy, infant nutrition, breastfeeding, hydrolyzed formulas, amino acid-based formulas, economic burden

## Abstract

Introduction: Cow’s milk protein allergy (CMPA) is one of the most common food allergies in early infancy and poses important clinical and economic challenges for affected children, their families, and healthcare systems. In Latin America, variability in diagnostic and therapeutic approaches remains substantial. Objective: We aim to systematically review the available evidence on CMPA, with emphasis on clinical characteristics, diagnosis, management, and economic impact, and to provide a complementary cost analysis of specialized formulas in the Colombian context. Methods: A systematic review was conducted according to PRISMA guidelines to synthesize current evidence on CMPA in pediatric populations. Studies published between 2010 and 2023 were screened using predefined eligibility criteria, and 46 studies were included in the qualitative synthesis. A complementary cost analysis was also performed to estimate the six-month costs associated with specialized infant formulas in Colombia, based on average age-specific formula consumption and standardized 2025 market prices. Results: The reviewed evidence confirms that CMPA is a heterogeneous condition with variable clinical manifestations and persistent diagnostic challenges, particularly in non-IgE-mediated presentations. Elimination of cow’s milk protein followed by oral food challenge remains the reference diagnostic approach. Breastfeeding with maternal dairy exclusion is consistently recommended as the preferred first-line strategy, whereas extensively hydrolyzed and amino-acid-based formulas are used when breastfeeding is not feasible or is insufficient. Estimated six-month costs ranged from COP 4,337,640 to COP 14,480,700 (approximately USD 1100–3600), depending on formula type. Conclusions: CMPA requires early recognition, careful clinical evaluation, individualized nutritional management, and improved access to effective and affordable treatment strategies.

## 1. Introduction

Food allergies represent an increasingly important public health problem worldwide. In the case of cow’s milk protein allergy (CMPA), a prevalence of approximately 0.6% up to 2014 and an estimated incidence of 2–3% during the first year of life have been reported [1]. More than 90% of food allergies in infants are related to foods such as cow’s milk, eggs, soy, peanuts, tree nuts, wheat, fish, and shellfish [2,3].

CMPA is the most frequent food allergy during the first months of life. In addition, it is associated with an increased risk of developing other atopic diseases, such as asthma, atopic dermatitis, and allergic rhinitis, within the so-called “atopic march” [4]. This condition affects the quality of life of patients and their families and leads to increased healthcare costs [4]. Although international guidelines for its management are available, important differences in diagnosis and treatment still persist in Latin America [1].

In many cases, CMPA is not recognized promptly by healthcare professionals, which delays both diagnosis and management [5,6].

The main objective of this study was to conduct a systematic review of the available evidence on CMPA, with emphasis on the Latin American context. As a complementary component, an estimation of the costs associated with the use of specialized formulas during the first six months of life in the Colombian context was performed.

Recent advances in the understanding of CMPA have focused on improved phenotypic classification, recognition of non-IgE-mediated forms, refinement of elimination and challenge-based diagnostic strategies, and the role of extensively hydrolyzed and amino-acid-based formulas in the induction of immune tolerance. The most recent international guidelines highlight breastfeeding combined with a maternal elimination diet as the preferred therapeutic approach, together with early recognition of the condition to reduce clinical complications and the associated economic burden.

## 2. Search Methods

### 2.1. Systematic Review

A systematic review of CMPA was conducted, accompanied by a complementary analysis of the costs associated with the use of infant formulas containing modified proteins as the sole source of nutrition for children aged 0 to 6 months.

Inclusion criteria comprised observational, descriptive, analytical, and epidemiological studies published between 2010 and 2023 involving pediatric populations. Studies focusing on non-standardized diagnostic techniques, alternative therapies, immunotherapy, or adult populations were excluded.

The systematic review was conducted in accordance with PRISMA 2020 guidelines. The review protocol was not prospectively registered. Searches were performed independently in PubMed, Frontiers, Clinical Key, NEJM, UpToDate, Pediatrics, and the Revista Colombiana de Gastroenterología. Search strategies combined MeSH terms and free-text keywords, including “cow’s milk protein allergy”, “milk allergy”, “food allergy in pediatrics”, and “food protein-induced enterocolitis syndrome (FPIES)”.

The review aimed to address the following research question: What are the clinical characteristics, diagnostic approaches, management strategies, and economic implications of CMPA in pediatric populations? Included studies were observational, clinical, analytical, descriptive, and epidemiological. Case reports relevant to the target population were also considered.

Each article was screened based on its title, abstract, and full text according to predefined eligibility criteria. The following search equations were used:

General: (“food allergy in pediatrics”) AND (“food allergies”).

Specific: (“cow’s milk protein allergy”), (“milk allergy”), (“FPIES”); (“composition of human and other mammalian milk”) AND (“allergic colitis and dermatitis”).

**Figure 1:**
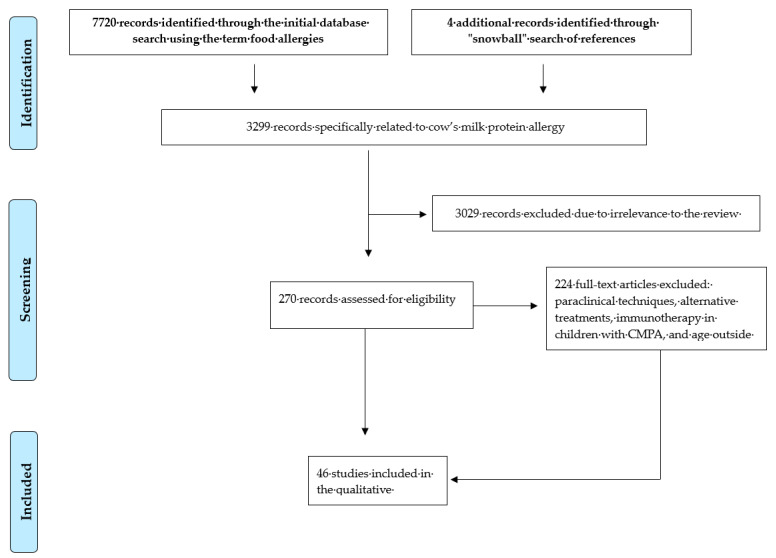
PRISMA flow diagram of the study selection process.

The initial database search identified 7724 records related to food allergies. After refining the search using CMPA-specific terms, 3299 records were retained. Subsequently, articles were screened for thematic relevance, focusing on the epidemiology, clinical manifestations, diagnostic approaches, and treatment of CMPA. Studies centered on non-standardized paraclinical techniques, alternative therapies, immunotherapy, or patient populations outside the predefined age range were excluded. Ultimately, 46 studies met the inclusion criteria and were selected for qualitative analysis (Figure 1).

### 2.2. Cost Analysis

Cost estimates were calculated based on the average monthly formula consumption by age, as recommended in pediatric feeding guidelines. Prices were obtained from commonly used pharmacies and distributors in Colombia and standardized to 2025 market values. Given the variability in market prices, minimum, maximum, and mean costs were reported to reflect cost fluctuations rather than to perform formal sensitivity analyses.

## 3. Results

### 3.1. Results of the Systematic Review

#### 3.1.1. Study Characteristics

CMPA is defined as an abnormal immune response that typically manifests during the first weeks of life after exposure to formulas containing cow’s milk proteins. Although it may also occur in exclusively breastfed infants, symptoms in this population tend to be milder [1,2,3,4,5]. CMPA can be classified into three types according to the underlying immune mechanism: IgE-mediated, non-IgE-mediated, and mixed [6]. These responses are triggered by early exposure to bovine milk proteins, either through maternal consumption transferred via breast milk or through the use of infant formulas containing cow’s milk derivatives [1].

In Latin America, available evidence indicates significant differences in the diagnostic and therapeutic approach to CMPA [2]. In Colombia, most studies have focused primarily on food allergies, often grouping CMPA within a broader category [2,7,8,9,10].

The largest descriptive study conducted in Colombia on gastrointestinal manifestations of food allergies identified cow’s milk protein as the most frequent allergen in the pediatric population evaluated [9]. Other reviewed studies confirm that cow’s milk is one of the most common allergens in early infancy [11]. However, the literature review reveals a lack of specific information on CMPA in the Colombian Caribbean region, highlighting the need to strengthen clinical and epidemiological research in this context.

Human milk is the preferred source of nutrition for infants, as it contains a variety of beneficial substances, including growth factors, peptides, hormones, prebiotics and probiotics, enzymes, cytokines, chemokines, as well as anti-inflammatory, antimicrobial, and antioxidant components [12,13]. It is a personalized biofluid that includes several bioactive and nutritional components. Among its proteins are caseins, which are present in α-, β-, γ-, and κ-isoforms. The predominant casein in human milk is β-casein (β-CN), which plays a role in improving calcium bioavailability. The most abundant whey proteins are immunoglobulin A (IgA, accounting for approximately 90%), lactoferrin, α-lactalbumin, and lysozyme [13,14]. The main carbohydrate is lactose, and the fats are primarily triglycerides, making up approximately 98% of the total lipid content [14].

Breastfeeding promotes the development of oral tolerance and may help prevent food allergies and atopic dermatitis [12] (Table 1). It exerts a protective effect against hypersensitivity development due to its lower content of foreign proteins, the presence of IgA (which provides passive protection against foreign proteins and pathogens), and soluble factors (e.g., prolactin) that may accelerate the maturation of the intestinal barrier and the infant’s immune response [15,16].

Regarding cow’s milk, its protein components include αS1-, αS2-, β-, and κ-caseins, as well as whey proteins such as α-lactalbumin (ALA), β-lactoglobulin (BLG), bovine lactoferrin, bovine serum albumin (BSA), and bovine immunoglobulins. Caseins and whey proteins represent approximately 80% and 20% of the total protein content, respectively. Most patients with cow’s milk protein allergy (CMPA) are sensitized to several of these proteins, including BLG, casein, ALA, BSA, bovine lactoferrin, and bovine immunoglobulins. These proteins may become less allergenic after heating; however, in the case of β-lactoglobulin, exposure to temperatures of approximately 121 °C for more than 20 min may actually increase its allergenicity [13,14,15]. Casein, BLG, and ALA are considered the main allergens in cow’s milk, and co-sensitization to more than one of these proteins is common [17] (Table 2).

The risk factors for this disease are not yet fully understood; however, it has been established that the risk of developing this condition is higher in individuals with a family history of atopy, especially when the maternal side is affected [3,4,5,6,7,8,9,10,11,12,13,14,15,16,17,18]. Not providing exclusive breastfeeding and introducing infant formula feeding may increase the risk of developing CMPA, as well as being born via cesarean section. In many healthcare institutions, due to hypoglycemia prevention policies and the increased frequency of cesarean delivery as a means of ending pregnancy, breastfeeding is delayed, and infant formula is introduced as the first feed. This practice has been shown to increase the risk of developing CMPA compared to controls who were fed with breast milk within the first 24 h [16,19,20,21,22].

Contrary to what might be expected—that prematurity increases the risk of developing this condition—evidence shows that these patients have a relatively low incidence [19].

This condition has been reported more frequently in infants from higher socioeconomic strata, although further studies are needed to clarify the strength of this association [23].

To understand the phenotypic manifestations of this condition, it is essential to recognize IgE-mediated reactions. IgE is highly prevalent in mucosal tissues and plays a central role in immunological memory, mediating rapid allergic responses that predominantly involve the skin, respiratory system, and gastrointestinal tract [6]. In contrast, non-IgE-mediated reactions involve type IV hypersensitivity, driven by delayed cellular responses, and are mainly associated with gastrointestinal symptoms. Mixed reactions, involving both mechanisms, have also been described [14,15,16].

In 82% of cases, the first signs and symptoms appear within the first four months of life, and in 95% of cases, they occur within the first year [1,2,3,4,5,24].

The clinical manifestations have been classified into three stages, based on the severity and timing of the reaction. Immediate reactions occur within the first 30 min after exposure and include dermatological symptoms such as urticaria, angioedema, and anaphylaxis, all of which are IgE-mediated. Intermediate (or delayed) reactions appear a few hours after ingestion of the allergen and are typically gastrointestinal, without IgE involvement. Late-onset reactions occur between one and five days after consumption and are primarily gastrointestinal, possibly accompanied by respiratory or cutaneous symptoms [5,8,24,25]. The role of an IgE-mediated response in these late-onset reactions remains uncertain [24,25,26] (Table 3). The involvement of more than one organ or system has been reported in 26% of patients [1,17,24].

The real challenge of this disease lies in its diagnosis, as it often requires time and multiple medical consultations before CMPA is suspected, diagnosed, and properly managed, which may cause stress and anxiety for patients and their families [22,27,28].

Based on a thorough medical history and physical examination, the diagnosis of CMPA is clinical, and no laboratory studies are required to confirm it [1]. Approximately a decade ago, the use of allergen elimination and observation of a favorable clinical response was considered sufficient to establish the diagnosis, without exposing the patient to additional risks [29]. However, this approach has evolved in recent years. Updated guidelines and meta-analyses now recommend that elimination and challenge tests are the most effective tools to diagnose non-IgE-mediated CMPA (non-IgE CMPA) [1,5,24,28,30,31,32]. The elimination test supports the initial clinical suspicion, while the reintroduction (oral food challenge) confirms the diagnosis [33,34].

In severe cases of food protein-induced enterocolitis syndrome (FPIES), re-exposure to cow’s milk protein may trigger a systemic reaction and should therefore be avoided [30,33]. It is recommended to eliminate cow’s milk protein from the diet, which typically leads to improvement and resolution of symptoms over variable time frames: 1–5 days in acute presentations, 1–2 weeks in cases of eczema or gastrointestinal bleeding, and up to 2–4 weeks in patients with constipation, diarrhea, and/or impaired nutritional status [1,30]. After symptom resolution, controlled reintroduction of cow’s milk protein (oral food challenge) is advised.

The test is considered positive when clinical symptoms reappear during the reintroduction phase. An alternative method is the Cow’s Milk-related Symptom Score (CoMiSS) questionnaire [18], which assesses the number and severity of symptoms potentially associated with CMPA. A score above 12 supports the diagnosis; however, it is a relatively time-consuming tool to administer.

When IgE-mediated reactions are suspected, specific IgE quantification, skin prick tests, or patch tests may be performed. None of these tools alone is diagnostic for CMPA, and some studies report low sensitivity [30,31,32,33,34,35]. A positive specific IgE result indicates sensitization but not necessarily clinical allergy, thus requiring clinical correlation and, in some cases, referral to allergy or immunology specialists [1,30].

Many patients undergo multiple consultations and are frequently misdiagnosed with lactose intolerance, infantile colic, dermatitis, or enterocolitis. While these are part of the differential diagnosis, CMPA should always be considered. Often, symptoms are treated in isolation without recognizing CMPA as a potential underlying cause, which may result in the unnecessary and permanent discontinuation of breastfeeding [36,37].

Finally, the treatment for children with CMPA consists of an elimination or exclusion diet until the patient develops oral tolerance to cow’s milk proteins, which is typically achieved after at least 6 months and no later than 3 to 4 years of age [19,34,38,39]. This approach involves completely removing all foods containing milk or its derivatives from the mother’s diet to prevent transfer through breast milk, as well as using protein-modified infant formulas [37].

**Figure 2:**
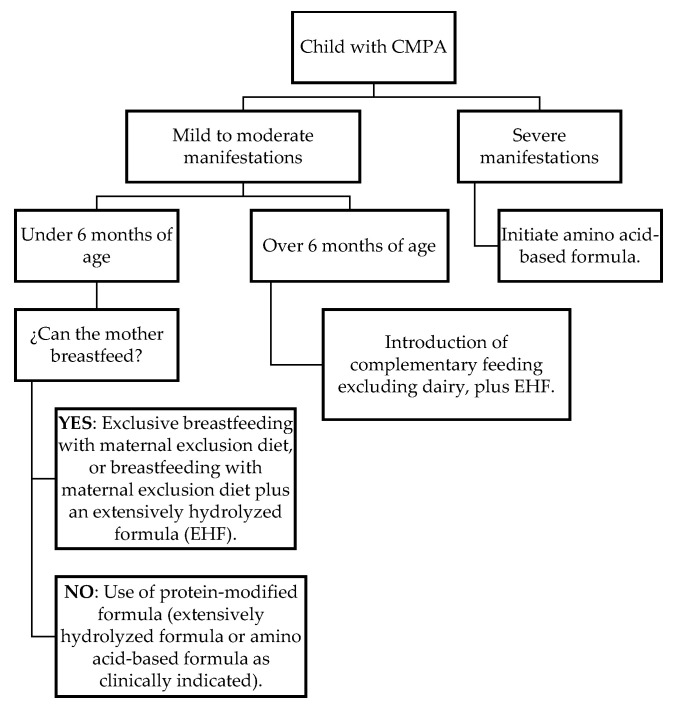
Management Algorithm According to Clinical Severity.

The elimination diet targets the adaptive immune system by suppressing the antigen-driven T-cell response [34,38]. In protein-modified formulas, their effectiveness lies in the absence of IgE-binding epitopes, which helps reduce allergic symptoms. Additionally, these formulas act on the intestinal mesenteric lymph nodes, increasing the number of regulatory T cells (Tregs), which are essential for inducing immune tolerance [4,28] (Figure 2).

Based on the most recent guidelines, with particular emphasis on American and European recommendations [1,5,24,28,31,32], we identify three management scenarios:

(1) Infants fed exclusively with breast milk: mothers who choose to provide exclusive breastfeeding should adhere to the elimination diet (Table 4) and should also receive supplementation with 1.0 g of calcium per day and 600 IU of vitamin D daily. There is no clinical evidence suggesting the need to exclude other proteins from the breastfeeding mother’s diet. In cases of suspected cross-reactivity with other foods, referral to allergy and nutrition specialists is recommended to evaluate the possibility of additional food allergies and to prevent maternal nutritional compromise.
(2) Infants fed with breast milk and protein-modified formula: this involves excluding cow’s milk protein from the maternal diet and substituting it with a protein-modified formula.
(3) Infants whose mothers cannot breastfeed: A protein-modified infant formula should be used. The choice of formula depends on the patient’s age, clinical condition, and the mother’s ability to breastfeed. Systematic removal of lactose in the management of CMPA is not supported and should only be undertaken in cases of transient intolerance due to enteropathy. Among the available protein-modified formulas are the following (Table 5).

Most guidelines recommend that extensively hydrolyzed formulas (EHFs) be used initially in the diagnostic elimination diet [1,18,24,28,32,34]. If clinical manifestations do not resolve within two weeks, it is recommended to discontinue EHFs and replace them with amino-acid-based formulas (AAFs). The patient should recover within 2 to 4 weeks. AAFs are considered 100% effective in controlling the clinical manifestations of CMPA [33]. Therefore, persistence of clinical manifestations during an elimination diet with AAFs should prompt further investigations to explain the symptoms and to rule out the diagnosis of CMPA [1].

#### 3.1.2. Duration of Management and Development of Tolerance

After completing the diagnostic process and initiating management, treatment should be maintained until the child develops oral tolerance, which typically occurs within 3–6 months for mild forms and proctocolitis, and 12–18 months for FPIES and severe forms [1,39,40]. In general, tolerance is expected by age 1 [35]. In cases of late-onset, IgE-mediated CMPA with cutaneous manifestations, among others, the allergy may be persistent and does not usually require additional allergy evaluation before age 1 [32].

#### 3.1.3. Oral Food Challenge for Tolerance Acquisition

Depending on the allergy mechanism and the child’s reaction history, the oral cow’s milk protein tolerance challenge can be performed either in a hospital setting or at home. This should be carried out gradually over approximately 4 weeks, ideally starting with baked products such as cookies or bread. In the second week, plain yogurt may be introduced, followed by cheeses, and finally small amounts of milk itself, with gradual increases (ideally up to 200 mL tolerated). If any reaction occurs, the challenge must be stopped immediately, and a specialist should be consulted [21,33].

It is important to clarify that after a prolonged exclusion period, children with immediate-type symptoms, personal history of atopy, or FPIES may develop an IgE-mediated mechanism to the excluded food [30]. Before performing the tolerance acquisition test in these cases, it is advisable to carry out a skin prick test or measure specific IgE to cow’s milk protein [10,40].

#### 3.1.4. Natural History and Persistence of CMPA

CMPA often resolves over time; however, this resolution is not always complete. Some children considered free of CMPA may retain a “residual disease,” preventing them from tolerating a “normal” intake of milk and dairy products later in life [32]. If CMPA persists beyond the first year of life, special formulas designed for that age group are required [30,41].

#### 3.1.5. Current Lack of Effective Prevention

Currently, no effective preventive measure for food allergy has been established [41]. Maternal dietary interventions during pregnancy or breastfeeding, the use of hydrolyzed formulas in at-risk infants, and the administration of probiotics or prebiotics have not shown sufficient evidence to prevent the development of CMPA [27,33].

#### 3.1.6. Economic Impact of CMPA Management

The economic impact of managing cow’s milk protein allergy can be significant and varies depending on multiple factors. Reviewed studies [42,43,44,45] analyzed in detail both the cost of protein-modified formulas and the overall economic impact, including medical care and laboratory testing. These studies conclude that establishing an early diagnostic suspicion, avoiding delays in the diagnostic process, and promoting breastfeeding with maternal protein exclusion as the best nutritional option for infants with CMPA are of vital importance.

In severe cases, and when breastfeeding is not a viable option, the use of these special formulas becomes necessary. However, this does not diminish their importance in the treatment and management of the allergy. Research emphasizes the need for early intervention and informed decision-making to reduce the economic impact and ensure the health and well-being of infants affected by cow’s milk protein allergy.

### 3.2. Cost Analysis of CMPA Management

In the cost analysis of patients who were fed exclusively with infant formula, it was determined that, based on a review of prices in the Colombian market in 2025, a healthy infant fed exclusively with stage 1 formula incurs an average cost of COP 60,014 per 375–400 g can, resulting in an estimated cumulative cost of COP 3,600,840 over the first six months of life.

In the case of protein-modified formulas designed for infants with CMPA, costs increase by up to four times compared with conventional formulas. An infant fed with extensively hydrolyzed formula (EHF) incurs a six-month cost of COP 4,337,640, while in other formulations this cost may reach COP 7,740,000 over the same period. Infants fed with amino-acid-based formula (AAF) incur a substantially higher cost, reaching COP 14,480,700 over six months (Tables S1 and S2).

Based on the cumulative six-month cost analysis, the estimated mean treatment cost per patient was COP 4,337,640 for partially hydrolyzed formulas (PHFs), COP 7,740,000 for extensively hydrolyzed formulas (EHFs), and COP 14,480,700 for amino-acid-based formulas (AAFs) (Table S3). To illustrate the potential distribution of treatment costs across formula types, aggregated six-month costs were estimated as COP 4,337,640 for PHF, COP 30,960,000 for EHF, and COP 28,961,400 for AAF (Table S4). PHFs were included only as a comparative cost reference and not as a recommended therapeutic option for CMPA.

## 4. Discussion

Systematic reviews that comprehensively address CMPA remain limited. Most published systematic reviews and meta-analyses focus on specific aspects of CMPA, including the efficacy and safety of hydrolyzed and amino-acid-based formulas, as well as the use of probiotics in infant formulas [46,47,48]. The importance of breastfeeding as both a protective and therapeutic factor has also been consistently emphasized [48]. In this context, the present review integrates pathophysiological, clinical, diagnostic, and therapeutic aspects of CMPA that have not always been addressed together in previous systematic reviews.

Both the Consensus on the Diagnosis and Treatment of Cow’s Milk Protein Allergy of the Latin American Society of Gastroenterology, Hepatology, and Nutrition [1] and other reviewed guidelines [1,5,10,24,30,34] agree that the reference diagnostic approach for suspected non-IgE-mediated CMPA is elimination of cow’s milk protein followed by an oral food challenge. However, some authors suggest reserving the elimination–challenge protocol for cases in which diagnostic uncertainty persists [28]. In addition, when IgE-mediated reactions are suspected, serum IgE testing may be considered; however, its diagnostic utility remains limited. Díaz et al. (2022) [30], after evaluating 239 ELISA tests for IgE, reported high specificity (>90%) but low sensitivity (<60%), suggesting that these tests may be more useful for confirmation than for detection of CMPA.

Diagnosis and management are closely interrelated in CMPA, since elimination of cow’s milk protein from the infant’s diet and strict adherence to this intervention are essential for symptom control and clinical improvement [1]. Current recommendations consistently support breastfeeding with maternal exclusion of dairy products as the preferred first-line strategy whenever feasible [1,5,10,30,32]. When breastfeeding is not possible or is insufficient, extensively hydrolyzed formulas are generally recommended as the preferred alternative, whereas amino-acid-based formulas are reserved for severe cases or for patients who do not respond adequately to hydrolyzed formulas [39,40,41,42,49].

Consistent with current recommendations, nutritional management should be maintained throughout the elimination period, ideally with appropriate dietary monitoring to prevent nutritional deficiencies [1,5,10,30,32]. The reviewed literature also highlights that oral tolerance to cow’s milk protein is an important goal of treatment. For this reason, most guidelines recommend gradual reintroduction after an adequate elimination period, with close monitoring of tolerance to differently processed forms of cow’s milk protein, including baked or fermented products [25,40,41,42,43,44,45,46,47,48,49,50]. In patients with severe initial manifestations, oral food challenge should preferably be conducted in a hospital setting [41]. This individualized approach is particularly important because some children may later develop IgE-mediated mechanisms, in which case additional immunological testing may be warranted [1].

Persistence of CMPA beyond the first year of life has been reported in a minority of cases [50]. In general, prolonged persistence is considered a less favorable prognostic indicator, particularly when IgE-mediated mechanisms are involved or when CMPA coexists with other food allergies [40]. In addition, the reviewed literature suggests that early allergic inflammation may be associated with the later development of functional gastrointestinal disorders, including constipation, irritable bowel syndrome, and functional abdominal pain [18].

The treatment of CMPA in Colombia also involves an important administrative component, as access to protein-modified formulas generally requires authorization through the MIPRES system of the Ministry of Health, followed by approval and distribution by the corresponding health insurer. Although these products are covered by the public health system, their use represents a substantial economic burden.

An important contribution of this manuscript is the complementary analysis of treatment costs associated with protein-modified formulas during the first six months of life. Mean per-patient costs ranged from COP 4,337,640 for partially hydrolyzed formulas (PHFs) to COP 7,740,000 for extensively hydrolyzed formulas (EHFs), reaching COP 14,480,700 for amino-acid-based formulas (AAFs). These figures indicate a markedly greater economic burden than breastfeeding with maternal exclusion diets or the use of conventional infant formulas. It should be noted that partially hydrolyzed formulas were included only for comparative economic purposes, since they are not recommended for the treatment of CMPA according to current guidelines.

The economic impact of CMPA extends beyond the direct cost of formula acquisition. Additional expenses include repeated medical consultations, diagnostic procedures, nutritional follow-up, dietary modifications, and the purchase of specialized or alternative foods. Indirect costs, such as caregiver time and loss of work productivity, may further increase this burden. Similar patterns have been reported internationally. For example, López et al. [49] reported six-month costs of MXN 19,080 for partially hydrolyzed formula in Mexico, while Sladkevicius [43] estimated that the management of 18,270 South African infants with CMPA during the first year of life amounted to 22.1 million Rands.

Taken together, these findings underscore the need to address CMPA not only as a clinical problem but also as an economic and public health issue. Improving access to timely diagnosis, appropriate nutritional management, and effective treatment alternatives is essential, as is strengthening education and awareness among healthcare professionals and families. At the same time, the complementary cost analysis presented here highlights the importance of considering the financial implications of dietary treatment, particularly in resource-constrained settings.

This review has some limitations. The available literature is heterogeneous in terms of study design, diagnostic criteria, and reported outcomes, which limits direct comparison across studies. In addition, the complementary cost analysis was based on estimated formula consumption and standardized market prices; therefore, it should be interpreted as an approximation of economic burden rather than as a formal cost-effectiveness evaluation. Nevertheless, the findings provide useful contextual evidence and support the need for future prospective studies that integrate both clinical and economic dimensions of CMPA.

## 5. Conclusions

CMPA is a clinically important condition that continues to pose diagnostic and therapeutic challenges because of its heterogeneous presentation and the limited availability of standardized diagnostic tools. The reviewed evidence supports breastfeeding with maternal dairy exclusion as the preferred first-line strategy, while specialized formulas remain an effective alternative when breastfeeding is not feasible or is insufficient. However, the complementary cost analysis highlights the substantial economic burden associated with these products in the Colombian context. These findings reinforce the need for timely diagnosis, standardized management, and improved access to effective and affordable nutritional treatment strategies.

## Figures and Tables

**Table 1:** Composition of Cow’s Milk and Human Milk.

Component	Human Milk	Cow’s Milk
Water (%)	87.6	87.6
Osmolarity (mOsm)	287–293	350
Energy (kcal/100 mL)	62–70	68
Lactose (g/L)	63–70	44–66
Proteins (g/L)	9–19	30–39
Fat (g/L)	21–40	33–54
Total Casein (g/L)	2.4–4.2	24.6–28
α-Casein	0.9–1.9	3.0–3.9
β-Casein	3.87	8.6–11
Total Whey Proteins (g/L)	6.2–8.3	5.5–7
α-Lactalbumin	1.9–3.4	1.0–1.5
β-Lactoglobulin	Absent	3.2–4
Casein/Whey Protein Ratio (%)	60/40	80/20
Lysozyme	0.04–0.2	Trace
Fatty Acids (% of total fatty acids)		
Saturated Fatty Acids (SFA)	39.4–45	55.7–72.8
Monounsaturated Fatty Acids (MUFA)	33.2–45.1	22.7–30.3
Polyunsaturated Fatty Acids (PUFA)	8.1–19.1	2.4–6.3
Linoleic Acid (C18:2)	6.0–17.7	1.2–3
Linolenic Acid (C18:3)	0.6–3.4	0.3–1.8
Omega-6/Omega-3 fatty acids (n-6/n-3)	7.4–8.1	2.1–3.7

Source: adapted from Cimmino et al., 2023 [13].

**Table 2:** Main Cow’s Milk Proteins Associated with Cow’s Milk Protein Allergy.

Protein	Allergen	Sensitization Rate in CMPA (%)	Cross-Reactivity	Characteristics
Caseins	αS1-casein (Bos d 9)	98	>85% with goat and sheep milk	Major allergen; heat-stable
	αS2-casein (Bos d 10)	94		
	β-casein (Bos d 11)	91		
	κ-casein (Bos d 12)	91		
Whey proteins	α-lactalbumin (Bos d 4)	51		Major allergen; lysozyme superfamily
	β-lactoglobulin (Bos d 5)	61		Major allergen; lipocalin family; heat-labile; most abundant whey protein; absent in human milk
Serum albumin	Bovine serum albumin (Bos d 6)	43	15–20% with beef	
Immunoglobulins	Bos d 7	36		
Lactoferrin	—	35		

Source: adapted from de Armentia et al., 2018 [17].

**Table 3:** Clinical manifestations by organ systems.

System Involved	Symptoms and Characteristics
Dermatological (30–70%)	Atopic dermatitis, urticaria, pruritus, erythema, morbilliform rash, perianal erythema.
Gastrointestinal (50–60%)	Hemorrhagic proctocolitis: Mucous and bloody stools, straining, and anal inflammation. Typically occurs before 3 months of age. Enteropathy: Diarrhea, steatorrhea, poor weight gain, abdominal distension, irritability, colic, constipation. Gastroesophageal reflux, vomiting, feeding refusal. Usually presents before 2 years of age.
Food Protein-Induced Enterocolitis Syndrome (FPIES)	Onset usually before 9 months of age. Major criterion: Vomiting 1–4 h after ingestion of cow’s milk protein (CMP) without typical IgE-mediated skin or respiratory symptoms. Minor criteria: (1) ≥1 repeat episode of vomiting after CMP ingestion (2) Recurrent vomiting 1–4 h after ingestion of another food (3) Lethargy (4) Marked pallor (5) Need for emergency care (6) Requirement for IV fluid support (7) Diarrhea within 24 h (usually 5–10 h) after CMP ingestion (8) Hypotension (9) Hypothermia
Respiratory (20–30%)	Rhinoconjunctivitis, bronchospasm, laryngospasm, asthma, cyanosis, chronic cough, pneumonias, apnea, sudden infant death syndrome (SIDS), Heiner’s syndrome.
Systemic	Anemia, anaphylactic shock. Growth and height delay may occur due to delayed diagnosis and persistent exposure to allergens.

Source: own elaboration, based on Toca, M.C., et al. (2022) [1].

**Table 4:** Dairy Exclusion Diet for Breastfeeding Mothers.

Category	Foods to Exclude (Examples)
Milk and dairy products	Whole milk, lactose-free milk, skim milk
Fermented dairy products	Yogurt, sour cream, heavy cream
Dairy fats	Butter, artificial butter flavor
Cheese products	Cheese, curd, cottage cheese
Milk derivatives	Whey, milk-derived prebiotics and probiotics
Dairy-based desserts and sweets	Ice cream, dulce de leche, cakes, nougat
Baked goods containing milk	Bread, cookies
Processed foods containing dairy	Certain hams, chocolates, sauces

Note: Food labels should be carefully checked before consumption, and cross-contamination during food preparation should be avoided—source: own elaboration, based on Rodríguez et al., 2023 [37].

**Table 5:** Infant Formulas Recommended and Not Recommended for the Management of CMPA.

Infant Formulas Recommended for CMPA	
Formula Type	Description/Indications
Extensively Hydrolyzed Formulas (EHFs)	The protein source is whey or casein. More than 85% of peptides have a molecular weight below 1500 Da. Palatability depends on the protein source, degree of hydrolysis, and lactose content. EHFs are first-line formulas for CMPA and are effective in over 90% of patients with mild-to-moderate symptoms. Rice-based EHFs may be considered when there is no response to other EHFs; however, they are not first-line options.
Amino-Acid-Based Formulas (AAFs)	These formulas contain no cow’s-milk-derived proteins and are considered 100% effective in CMPA. They are recommended when: (i) no response to EHFs; (ii) multiple food allergies; (iii) severe CMPA (anemia, hypoalbuminemia, malnutrition); (iv) persistent symptoms in exclusively breastfed infants despite maternal dietary restrictions; (v) eosinophilic esophagitis; (vi) FPIES, anaphylaxis, or Heiner syndrome.
Infant Formulas Not Recommended for CMPA	
Formula Type	Rationale
Partially Hydrolyzed Formulas (PHFs)	Peptides retain allergenic epitopes capable of triggering allergic reactions.
Soy-Based Formula and Plant-Based Beverages (almond, oat, etc.)	There is a risk of cross-reactivity with soy; they may be considered only in children over 6 months with IgE-mediated CMPA who are not exclusively breastfed. Plant-based beverages are not nutritionally adequate.

Source: own elaboration, adapted from Vandenplas et al., 2021 [38].

## Data Availability

The data presented in this study are available on reasonable request from the corresponding author.

## Supplementary Materials

The following supporting information can be downloaded at: https://www.mdpi.com/article/10.3390/diseases14040146/s1, Table S1: Summary of data used for the cost analysis, including indicative daily amounts, which are not mandatory for infants; Table S2: Prices of standard, partially hydrolyzed, extensively hydrolyzed, and amino acid-based infant formulas (400 g); Table S3: Cost of protein-modified formulas during the first 6 months in Colombia (2025); Table S4: Cumulative cost of CMPA management according to the formula used.

## References

[1] Toca M.C., Morais M.B., Vázquez-Frias R., Becker-Cuevas D.J., Boggio-Marzet C.G., Delgado-Carbajal L., Higuera-Carrillo M.M., Ladino L., Marchisone S., Messere G.C. (2022). Consenso sobre el diagnóstico y el tratamiento de la alergia a las proteínas de la leche de vaca de la Sociedad Latinoamericana de Gastroenterología, Hepatología y Nutrición. Rev. Gastroenterol. México.

[2] Velasco-Benítez C.A. (2012). Epidemiología de la alergia alimentaria en la edad pediátrica. Rev. Gastrohnup Año.

[3] Nwaru B.I., Hickstein L., Panesar S.S., Roberts G., Muraro A., Sheikh A., EAACI Food Allergy Anaphylaxis Guidelines, Group (2014). Prevalence of common food allergies in Europe: A systematic review and meta-analysis. Allergy.

[4] Zuluaga Velásquez L.C., Ramírez Rodríguez N., Mejía Pérez L.K., Vera Chamorro J.F. (2018). Desenlaces del tratamiento con una fórmula extensamente hidrolizada a base de suero en lactantes con alergia a la proteína de leche de vaca. Rev. Colomb. Gastroenterol..

[5] Cubides-Munevar A.M., Linero-Terán A.S., Saldarriaga-Vélez M.A., Umaña-Bautista E.J., Villamarín Betancourt E.A. (2020). Alergia a la proteína de leche de vaca. Enfoque Diagnóstico Y terapéutico. Rev. Colomb. Gastroenterol..

[6] Baillieau F. Inmunoglobulina E: Revisión y Actualización de su rol en la Salud y Enfermedad Immunoglobulin e: Review and Update Their Role in Health and Disease. Com.ar. http://adm.meducatium.com.ar/contenido/articulos/100500054_13/pdf/100500054.pdf.

[7] Mehaudy R., Parisi C.A.S., Petriz N., Eymann A., Jauregui M.B., Orsi M. (2018). Prevalencia de alergia a la proteína de la leche de vaca en niños en un hospital universitario de la comunidad. Arch. Argent. Pediatría.

[8] Vieira M.C., Morais M.B., Spolidoro J.V.N., Toporovski M.S., Cardoso A.L., Araujo G.T.B., Nudelman V., Fonseca M.C.M. (2010). A survey on clinical presentation and nutritional status of infants with suspected cow’s milk allergy. BMC Pediatr..

[9] Vista de Alergia Alimentaria Gastrointestinal: Prevalencia, Caracterización y Costos Directos en un Centro de Remisión en Bogotá. Revistagastrocol.com. https://revistagastrocol.com/index.php/rcg/article/view/789/1374.

[10] Cervantes-De La Torre K., Guillen Grima F., Aguinaga Ontoso I., Mendoza Mendoza A. (2018). Presencia de alergias en menores por consumo temprano de alimentos en Barranquilla, Colombia. Rev. Salud Pública.

[11] Laignelet Hernández H.H., Hernández Mantilla N. (2022). Alergia alimentaria gastrointestinal: Prevalencia, caracterización y costos directos en un centro de remisión en Bogotá. Rev. Colomb. Gastroenterol..

[12] Hatmal M.M., Al-Hatamleh M.A.I., Olaimat A.N., Alshaer W., Hasan H., Albakri K.A., Alkhafaji E., Issa N.N., Al-Holy M.A., Abderrahman S.M. (2022). Immunomodulatory properties of human breast milk: MicroRNA contents and potential epigenetic effects. Biomedicines.

[13] Cimmino F., Catapano A., Villano I., Di Maio G., Petrella L., Traina G., Pizzella A., Tudisco R., Cavaliere G. (2023). Invited review: Human, cow, and donkey milk comparison: Focus on metabolic effects. J. Dairy Sci..

[14] Sampson H.A., Feldman M., Friedman L.S., Brandt L.J. (2021). Alergias alimentarias. Sleisenger y Fordtran. Enfermedades Digestivas y Hepáticas: Fisiopatología, Diagnóstico y Tratamiento.

[15] Järvinen-Seppo K.M., Sicherer S.H., TePas E. (2022). Milk Allergy: Clinical Features and Diagnosis.

[16] Saad K., Ahmad A.R., El-Tellawy M.M., El-Ashry A.H., Abdelsalam E.M.N., Alruwaili T.A.M., Elhoufey A. (2020). Cow milk protein allergy: Clinical phenotype and risk factors. Curr. Trends Immunol..

[17] De Armentia S.L.L. Alergia a Proteínas de Leche de Vaca. Pediatriaintegral.es. https://www.pediatriaintegral.es/wp-content/uploads/2018/xxii02/02/n2-076-086_SantiLapena.pdf.

[18] Pensabene L., Salvatore S., D’Auria E., Parisi F., Concolino D., Borrelli O., Thapar N., Staiano A., Vandenplas Y., Saps M. (2018). Cow’s milk protein allergy in infancy: A risk factor for functional gastrointestinal disorders in children?. Nutrients.

[19] Yanan J., Yan X. (2021). Avances en el diagnóstico y tratamiento de la alergia a la proteína de la leche de vaca en bebés prematuros. Rev. China Med. Prev..

[20] Chu S., Zhang Y., Jiang Y., Sun W., Zhu Q., Wang B., Jiang F., Zhang J. (2017). Cesarean section without medical indication and risks of childhood allergic disorder, attenuated by breastfeeding. Sci. Rep..

[21] Yang X., Zhou C., Guo C., Wang J., Chen I., Wen S.W., Krewski D., Yue L., Xie R.-H. (2022). The prevalence of food allergy in cesarean-born children aged 0–3 years: A systematic review and meta-analysis of cohort studies. Front. Pediatr..

[22] Mehaudy R., Jáuregui M.B., Vinderola G., Guzmán L., Martínez J., Orsi M., Parisi C.A. (2022). Alergia a la proteína de la leche de vaca; nuevos conocimientos desde una visión multidisciplinaria. Arch. Argent. Pediatr..

[23] Arancibia M.E., Lucero Y., Miquel I., Marchant P., Rodriguez L., Alliende F., Ríos G., Maturana A. (2020). Association of cow’s milk protein allergy prevalence with socioeconomic status in a cohort of Chilean infants. J. Pediatr. Gastroenterol. Nutr..

[24] Alergia a Las Proteínas de Leche de Vaca no Mediada por IgE Documento de Consenso de la Sociedad Española de Gastroenterología, Hepatología y Nutrición Pediátrica (SEGHNP), la Asociación Española de Pediatría de Atención Primaria (AEPap), la Sociedad Española de Pediatría Extrahospitalaria y Atención Primaria (SEPEAP) y la Sociedad Española de Inmunología Clínica, Alergología y Asma Pediátrica (SEICAP). https://www.seghnp.org/documentos/documento-de-consenso-sobre-alergia-proteinas-de-leche-de-vaca-no-mediada-por-ige.

[25] Navarro D., Arrieta A., López K., Belandria K., Quintana B., Enicar P., Figuereo C., Rossell A., Nogales A. (2013). Desarrollo de tolerancia oral en niños con alergia a la proteína de leche de vaca: Seguimiento de 10 años. Gen.

[26] Tang R., Lyu X., Liu Y., Zhu M., Yang X., Wu Z., Han B., Wu S., Sun J. (2022). Four clinical phenotypes of cow’s milk protein allergy based on dairy product specific IgE antibody types in North China. Front. Immunol..

[27] Cuevas Rivas A.P., Iglesias Leboreiro J., Bernárdez Zapata I., Martina Luna M., Venegas Andrade A., Koretzky S.G. (2020). Diagnóstico y tratamiento de la alergia a proteínas de la leche de vaca en un hospital privado de la Ciudad de México. Arch. Investig. Matern. Infant..

[28] Bagés M.C., Chinchilla Mejía C.F., Ortíz Piedrahita C., Plata García C.E., Puello Mendoza E.M., Quintero Hernández O.J., Riveros López J.P., Sosa Giraldo F.J., Wilches Luna A., Vera Chamorro J.F. (2020). Recomendaciones sobre diagnóstico y tratamiento de la alergia a la proteína de la leche de vaca en población pediátrica colombiana. Posición de expertos. Rev. Colomb. Gastroenterol..

[29] Cervantes Bustamante R., Cervantes R., María D., Sánchez P., Bacarreza D., Erika D., Barrios M., Flora D., Mondragón Z., Mata N. Artículos de Revisión. https://www.medigraphic.com/pdfs/revenfinfped/eip-2007/eip074f.pdf.

[30] Díaz M.C., Lavrut A.J., Slullitel P., Souza M.V. (2022). Usefulness of analytic tests for the diagnosis of cow’s milk protein allergy. Arch. Argent. Pediatría.

[31] Dupont C., Chouraqui J.P., Linglart A., Bocquet A., Darmaun D., Feillet F., Frelut M.L., Girardet J.P., Hankard R., Rozé J.C. (2018). Nutritional management of cow’s milk allergy in children: An update. Arch. Pediatr..

[32] Allen H.I., Pendower U., Santer M., Groetch M., Cohen M., Murch S.H., Williams H.C., Munblit D., Katz Y., Gupta N. (2022). Detection and management of milk allergy: Delphi consensus study. Clin. Exp. Allergy.

[33] Sociedad Chilena de Alergia e Inmunología (SCAI) (2021). Guía Clínica Alergia a la Proteína de Leche de Vaca (APLV).

[34] García Mérida M.J., Espín Jaime B., AEPap (2020). Alergia a las proteínas de la leche de vaca no mediada por IgE. Congreso de Actualización Pediatría 2020.

[35] Linhart B., Freidl R., Elisyutina O., Khaitov M., Karaulov A., Valenta R. (2019). Molecular Approaches for Diagnosis, Therapy and Prevention of Cow’s Milk Allergy. Nutrients.

[36] Vargas-Zarate M., Becerra-Bulla F., Balsero-Oyuela S.Y., Meneses-Burbano Y.S. (2020). Lactancia materna: Mitos y verdades. Artículo de revisión. Rev. Fac. Med..

[37] Rodríguez González M., Mendoza-Hernández D.A. (2016). Dieta de exclusión de leche de vaca... ¿cómo?. Alerg. Asma Inmunol. Pediatr..

[38] D’Auria E., Salvatore S., Pozzi E., Mantegazza C., Sartorio M.U.A., Pensabene L., Baldassarre M.E., Agosti M., Vandenplas Y., Zuccotti G. (2019). Cow’s milk allergy: Immunomodulation by dietary intervention. Nutrients.

[39] D’Auria E., Salvatore S., Acunzo M., Peroni D., Pendezza E., Di Profio E., Fiore G., Zuccotti G.V., Verduci E. (2021). Hydrolysed Formulas in the Management of Cow’s Milk Allergy: New Insights, Pitfalls and Tips. Nutrients.

[40] Sackesen C., Altintas D.U., Bingol A., Bingol G., Buyuktiryaki B., Demir E., Kansu A., Kuloglu Z., Tamay Z., Sekerel B.E. (2019). Current trends in tolerance induction in cow’s milk allergy: From passive to proactive strategies. Front. Pediatr..

[41] López L.L.C. Impacto en la Economía Familiar por uso de Sucedáneos de Leche Materna en Bebés Sanos y uso de Fórmulas Especiales 2010. Medigraphic.com. https://www.medigraphic.com/pdfs/conapeme/pm-2010/pm101e.pdf.

[42] Sladkevicius E., Guest J.F. (2010). Modelling the health economic impact of managing cow milk allergy in South Africa. J. Med. Econ..

[43] Sladkevicius E., Guest J.F. (2010). Budget impact of managing cow milk allergy in the Netherlands. J. Med. Econ..

[44] Cawood A.L., Meyer R., Grimshaw K.E., Sorensen K., Acosta-Mena D., Stratton R.J. (2022). The health economic impact of cow’s milk allergy in childhood: A retrospective cohort study. Clin. Transl. Allergy.

[45] Sorensen K., Cawood A.L., Gibson G.R., Cooke L.H., Stratton R.J. (2021). Amino Acid Formula Containing Synbiotics in Infants with Cow’s Milk Protein Allergy: A Systematic Review and Meta-Analysis. Nutrients.

[46] Qamer S., Deshmukh M., Patole S. (2019). Probiotics for cow’s milk protein allergy: A systematic review of randomized controlled trials. Eur. J. Pediatr..

[47] Poddar U., Yachha S.K., Krishnani N., Srivastava A. (2010). Cow’s milk protein allergy: An entity for recognition in developing countries. J. Gastroenterol. Hepatol..

[48] Turner P.J. (2013). Persistent allergy to cow’s milk: Of greater a clinical concern than other food allergies. Pediatr. Allergy Immunol..

[49] Stróżyk A., Horvath A., Meyer R., Szajewska H. (2020). Efficacy and safety of hydrolyzed formulas for cow’s milk allergy management: A systematic review of randomized controlled trials. Clin. Exp. Allergy.

[50] Topal E., Çeliksoy M.H., Arga M., Kaynak M.S., Duman Y., Demirtaş S., Alataş C., Tonbul H., Ökmen Z.H., Dalkılıç H.M. (2019). Independent predictive factors for the persistence and tolerance of cow’s milk allergy. Int. Forum Allergy Rhinol..

