# Supplementary material for: Cow’s Milk Protein Allergy, a Systematic Review of Clinical Characteristics, Diagnosis, Management, and Economic Impact
Source: Diseases. 2026 Apr 17;14(4):146. doi: 10.3390/diseases14040146 (PMC13115248; doi:10.3390/diseases14040146)
Supplement: Supplementary file 1 [file diseases-14-00146-s001.zip › diseases-4019971-supplementary.pdf]

diseases

Systematic Review

Cow's Milk Protein Allergy, a Systematic Review of Clinical Characteristics, Diagnosis, Management, and Economic Impact

Fabiola Menco Contreras [1], Karina Pastor-Sierra [2,*] and Nany Castilla Herrera [1]

[1] Especialista en Pediatría, Facultad de Ciencias de la Salud, Universidad del Sinú E.B.Z., Montería 230001, Colombia; fabiolamenco@unisinu.edu.co (F.M.C.); nanycastilla@unisinu.edu.co (N.C.H.)
[2] Grupo de Investigación Biomédicas y Biología Molecular, Facultad de Ciencias de la Salud, Universidad del Sinú E.B.Z., Montería 230001, Colombia.
* Correspondence: karinapastor@unisinu.edu.co; Tel.: +57 3233651725

Table S1. Summary of data used for the cost analysis; indicative daily amounts, but not mandatory for an infan.

Age	Number of feeds in 24 hours	Total volume in 24 hours (cc)	Number of cans (400 g)
0–1 months	7–9 feeds of 2 ounces	413–531 cc	6–8 cans
2–3 months	5–7 feeds of 4–5 ounces	590–1,032 cc	8–12 cans
3–4 months	5–6 feeds of 6–7 ounces	885–1,236 cc	12 cans
5–6 months	5 feeds of 6–7 ounces, considering initiation of complementary feeding	885–1,030 cc	9–12 cans

1 ounce: 29.5 cc: One formula scoop is equivalent to approximately 4–4.5 g of powdered formula.
Source: Adapted from Cuevas López, 2010 [25].

Table S2. Prices of Standard, Partially Hydrolyzed, Extensively Hydrolyzed, and Amino Acid–Based Infant Formulas (400 g).

Commercial name of stage-1 formula	Price per 400-g can (Colombia pesos)
Nutriben 1	$ 64,963
Alula Gold	$ 80,925
NAN supreme pro	$ 87.800
NAN optipro	$ 68.300
Nestogeno	$ 45,324
Similac 1	$ 56,746
Baby klim 1	$ 38,577
Infacare	$ 45,658

Blemil 1	$ 54,514
Alpina baby 1	$ 44,633
Nutribaby	$ 61,412
Enfamil con hierro	$ 27.950
Enfamil 1	$ 84,700
Enfamil promental	$60.014
Average	$60.014
Maximum	$87.800
Minimum	$27.950
**Commercial name of FPH**	
NAN expert pro confort	$ 71,015
Nutriben comfort	$ 103,000
Similac total confort	$ 62,000
Alula Gold confort	$ 80,000
Blemil confort	$ 67,040
Nestogeno confort total	$ 52,000
Enfamil confort	$ 71,000
Average	$ 72,294
Maximum	$ 103,000
Minimum	$ 52,000
**Commercial name of FEH**	
Nutrilon pepti junior	$ 155,000
Nutrilon peptisineo	$ 135,000
Althera	$ 121,900
Nutramigen con LGG	$ 133,600
Alfare	$ 131,500
Average	$ 129,788
Maximum	$ 155,000
Minimum	$ 121,900
**Commercial name of FAA**	
Neocate LCP	$ 251,600
Puramino	$ 265,000
Elecare	$ 177,100
Alfamino	$ 271,678
Average	$241,345
Maximum	$271,678
Minimum	$177,100

Source: Authors' elaboration, based on current prices in Colombia in 2025, different pharmacies and available points of sale were taken into account.

diseases

Table S3. Cost of Protein-Modified Formulas During First 6 Months (Colombia, 2025).

Formula Type	Min (COP)	Max (COP)	Mean (COP)
PHF	$3.120.000	$6.180.000	$4.337.640
EHF	$5.889.000	$9.300.000	$7.740.000
AAF	$10.626.000	$16.300.680	$14.480.700

CMPA: Cow's milk protein allergy; **AAF:** Amino acid–based formula; **EHF:** Extensively hydrolyzed formula; **PHF:** Partially hydrolyzed formula. Source: Authors' elaboration, based on current prices in Colombia (2025).

Table S4. Cumulative Cost of CMPA Management by Formula Used.

Formula Type	n (%)	Total Cost (COP)
PHF	14.2 %	$ 4.337.640
EHF	57.1%	$ 30.960.000
AAF	28.5 %	$ 28.961.400

CMPA: Cow's milk protein allergy; **AAF**: Amino acid–based formula; **EHF:** Extensively hydrolyzed formula; **PHF**: Partially hydrolyzed formula. **Source:** Reviewed medical records.
